# Fatigue as a key factor for testing knee stability with single leg drop landing for injury prevention and return to play tests

**DOI:** 10.3389/fspor.2023.1243732

**Published:** 2023-11-06

**Authors:** S. Becker, S. Simon, C. Dindorf, J. Dully, E. Bartaguiz, L. Schmitz, N. Kothe, M. Fröhlich, O. Ludwig

**Affiliations:** Department of Sport Science, RPTU Kaiserslautern-Landau, Kaiserslautern, Germany

**Keywords:** neuromuscular control, dynamic knee valgus, sensorimotor system, SLDL, FPPA

## Abstract

**Objectives:**

Fatigue can decrease knee stability and increase the injury risk. However, fatigue is rarely being applied throughout movement analysis. The aim of this study was to investigate if the knee stability throughout SLDLs differ between cyclic and acyclic sports, before and after fatigue in general, and between the dominant and non-dominant leg of soccer players.

**Methods:**

A total of 43 active male (*n* = 34) and female (*n* = 9) athletes (age: 26.5 ± 7.2) participated in this study with a pre-post-design. Subjects performed a single leg drop landing (SLDL) from a plyobox. For each leg, the two-dimensional frontal plane projection angle (FPPA) was analyzed. After pretesting the shuttle run test was performed until exhaustion, before repeating the measurements.

**Results:**

ANOVA with repeated measures was applied and identified no significance difference for the FPPA between cyclic and acyclic sports (*F* = 0.98, *p* = 0.33), a significant difference before and after fatigue (*F* = 12.49, *p* = 0.002) and no significant difference between the dominant and non dominant leg of soccer players (*F* = 4.35, *p* = 0.26).

**Discussion:**

Fatigue seems to be able to have a significant influence on knee stability in the frontal axis. Therefore, fatigue should be included in motion analysis for injury prevention and return to play tests because during this physical state most injuries happen.

## 1. Introduction

Knee stability is a fundamental base for almost all sports and therefore desirable (1). Whether in acyclic movements (e.g., game sports) or cyclic movements (e.g., athletics), the knee joint is a sensitive structure and unfortunately too frequently affected by injury (2–4). Increased dynamic knee valgus is known to increase the risk of injury and joint stress, pointing to the dominant mechanism of injury to the anterior cruciate ligament (5).

Cyclic sports, such as running and cycling, involve repetitive movements and have relatively low demands on knee stability compared to acyclic sports (6), which involve more complex and unpredictable movements (7). In cyclic sports, the knee primarily moves in a single plane of motion with minimal lateral or rotational movements (8). The repetitive nature of these movements tends to strengthen the muscles that stabilize the knee joint in this more or less single plane (9). Acyclic sports, such as soccer, basketball, or tennis, require rapid changes in direction, jumping, and landing (6). In acyclic sports, the knee is subjected to multidirectional forces, which require greater stability and control (7). The movements in these sports involve lateral and rotational movements (10), which place higher demands on the knee joint and its surrounding muscles (8, 11). The knee must be able to absorb and transfer forces in multiple planes of motion, which requires the coordinated action of multiple muscle groups (9, 12).

Neuromuscular fatigue affects our sensorimotor system and can impair joint position sense, alter muscle activation patterns, and reduce muscle strength, all of which can lead to a decrease in knee stability and an increased risk of injury (13–15). It is important for athletes to manage physical shape in order to minimize the negative effects of neuromuscular fatigue throughout dynamic activities and competitions. On the other hand, both physicians and sports scientists, which use the single leg drop landing (SLDL) as a preventive knee stability testing procedure, should probably consider testing intentionally under fatigued conditions, since lots of injuries happen in a fatigued state (16, 17). Knee complaints and injuries affect all performance levels and statistically carry a particularly high prevalence. Many people associate diagnostic procedures, in the sense of movement analysis for quantifying knee stability, with professional sports in particular, but in the meantime, testing procedures have become established on the market that are affordable and economical, so that they have became of interest for all sports levels (18). This is why SLDL with two-dimensional, videometric support is an efficient tool for testing knee stability (17, 19).

On basis of the results of Ludwig et al. (20) the measurement procedure will be replicated and the influence of neuromuscular fatigue on the SLDL will be investigated additionally. Ludwig and colleagues identified a significant difference in knee stability between the dominant and non-dominant leg in adolescent soccer players (20). The sport-specific requirement, kicking with a dominant leg, and stabilizing with the other leg seems to be the reason. In contrast to the 14.6 years old athletes they worked with, it would be interesting to see, if those results would be reproducible in older athletes. Furthermore, it is known that statistically the probability of injury can increase with progressive load (21). Knee injuries (e.g., ACL-ruptur) and the change in the biomechanics of the knee are multifactorial and therefore prevention should also be multi-perspective (13). Those neurophysiological changes can translate into a joint control reduction. Nevertheless, reality shows that very few investigators pre-tire athletes before SLDLs are scheduled for prevention screenings (22). Therefore, the aim of this study is to answer the following questions:

(1) Does the knee stability throughout SLDLs differ between cyclic and acyclic sports?
(2) Is there a statistical difference in the frontal plane projection angle (FPPA) of the knee throughout a SLDL before and after fatigue?
(3) Is there a statistical difference between the frontal plane projection angle (FPPA) of the knee for the dominant and non-dominant leg throughout a SLDL before and after fatigue?

In contrast to previous studies using the SLDL, we examine whether there is a difference between cyclic and acyclic athletes and to what degree fatigue plays a role using the SLDL. Ultimately, we would like to check whether the results of Ludwig et al. (20) would generate similar results with the same conditions but older subjects.

## 2. Materials and methods

### 2.1. Subjects

The sample size was prior calculated using G*Power (Version 3.1.9.6 for Macintosh, University of Kiel, Germany) for a repeated ANOVA (between factors, *f* = 0.25, *α* = 0.05). A minimum group size of 40 persons was calculated (power 0.96), which was increased to 43 (9 women, 34 men) due to expected dropouts, which didn't occur. The study was based and carried out in accordance with the current guidelines of Declaration of Helsinki and was approved by the responsible ethics commission (No. 55). All participants signed informed consent forms, including the permission to publish the results. The authors have no conflict of interest to declare.

All subjects were active athletes and came from an acyclic sport (*n* = 27; game sports and combat sports) or a cyclic individual sport (*n* = 16; athletics and cycling). For the comparison of side differences between the dominant leg (kicking leg) and non-dominant leg (stance leg) only the soccer players (*n* = 16) were included.

Exclusion criteria were athletes >35 years, illness, acute complaints or injuries, previous injuries (e.g., ACL injury, meniscal injuries) and two-footedness for soccer players for the comparison of the dominant and non-dominant leg.

### 2.2. Test procedure

Test subjects came in a rested state without intensive physical activity 48 h before the measurements. Further, they came to the study wearing tight, short sportswear and performed the trials torso-free or in a sports bra. The SLDLs were performed barefoot to exclude the influence of the shoe, which can be both stabilizing or destabilizing. Three markers with a diameter of 8 mm were fixed at three anatomical landmarks: (1) anterior superior iliac spine (ASIS), (2) center of the patella (CP), (3) middle of the connecting line between the lateral and medial malleoli (MM). Only one investigator palpated the anatomical landmarks and fixed the markers. Before the pretest, the neutral leg axis was recorded by positioning the participants in a hip-width stance in front of the camera (4 K and 60 FPS). The camera was placed 250 cm orthogonal to the edge of the box at a height of 85 cm. Participants were then instructed in a standardized manner for the SLDL. The SLDL was performed from a 35 cm high box (PlyoBox). The arms were crossed behind the back to decouple the potentially stabilizing influence of the arms, thus focusing on the actual muscles for stabilizing the leg axis. The SLDL was initiated with a double-legged forward jump, landing on one foot only. A landing zone of 50 × 50 cm was marked immediately in front of the box to visualize the landing zone. Each subject was allowed to make three test jumps and was verbally corrected if necessary. Only one valid shot was then taken for each leg to reduce learning effects and to replicate the study (20). A trial was considered invalid if there was balance disturbance during landing (severely lateralized trunk tilt), arms were disengaged from the hips, or foot positioning on the ground changed during the landing process (extra hops for balance). Subsequently, subjects underwent the shuttle run until exertion for introducing fatigue before repeating the same procedure for the post-test with a delay of approximately four minutes (location change, maker check).

**Figure 1:**
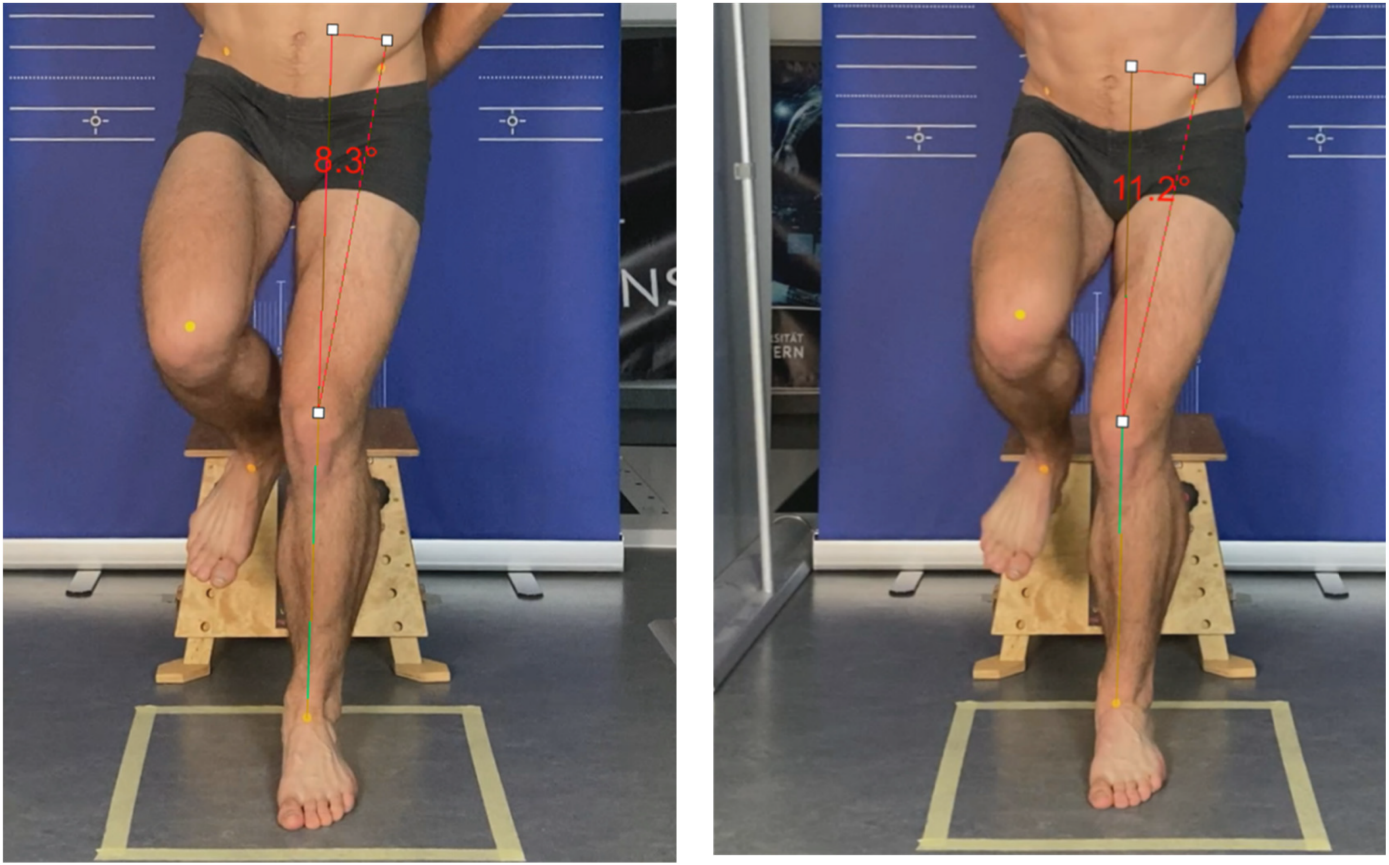
Comparison of pre (left) and post (right) fatigue by the frontal plane projection angle (FPPA) with a difference of 2.9°.

Knee stability after the SLDL is evaluated using the front plane projection angle (FPPA). This measures the intersection angle between the tibia and femur in the frontal view (see Figure 1). FPPA has been shown to be a valid parameter in previous studies. The kinematic angle evaluation was performed with Darfish 8.0 Pro Suite (Darfish, Fribourg, Switzerland). The evaluation of the FPPA was based on the normative data by Herrington and Munro (23). For the unilateral landing task the FPPA should be in the range of 5–12° for females and 1–9° for males.

### 2.3. Fatigue

In order to simulate muscular loading, the test persons had to run the shuttle run test according to Leger et al. (24) between the pre-test and the post-test. This is a standardized test procedure for measuring anaerobic endurance capacity (25). For this purpose, the test persons ran back and forth over a distance of 20 m with a change of direction. The running speed was controlled by an acoustic signal and increased successively. The test starts at 8.5 km/h and becomes faster by 0.5 km/h every minute. One subject always runs individually and until individual subjective exertion. At the end of the test, the subjective sensation was queried via the RPE scale (26) and the heart rate was recorded using a Polar FT7 (Polar Electro, Kempele, Finnland).

### 2.4. Statistics

Statistics were calculated with IBM SPSS (29.0 for Macintosh, Chicago, IL, USA). The results are stated as mean values ± standard deviations and 95% confidence intervals. To analyze pre-post-effects of fatigue and group differences between cyclic and acyclic sports an ANOVA with repeated measures was applied. Additionally, to analyze the pre-post-effects of fatigue and differences between the dominant and non-dominant leg within acyclic sports another ANOVA with repeated measures was applied. All test requirements (normal distribution, variance homogeneity) were checked and confirmed. Significance level was set to *p* < 0.05. Partial Eta Squared (np^2^) is provided as a effect size for significant results (.01 = small effect; 0.06 = medium effect, .14 = large effect).

## 3. Results

Table 1 shows anthropometric data and further details of the 43 test persons.

**Table 1:** Anthropometric data, sport experience, sport volume and loading of the 43 test persons (mean ± standard deviation).

	Overall (*n* = 43)	Cyclic (*n* = 16)	Acyclic (*n* = 27)	DOM vs. nDOM (*n* = 16)
Age [years]	26.5 ± 7.2	25.6 ± 3.0	24.8 ± 2.9	25.1 ± 3.4
Height [cm]	179.6 ± 7.7	176.4 ± 5.9	181.6 ± 7.0	183.2 ± 4.5
Weight [kg]	77.2 ± 13.1	72.6 ± 8.4	79.9 ± 14.3	81.3 ± 11.5
Sport experience^a^ [years]	10.1 ± 7.5	4.9 ± 1.4	13.2 ± 7.8	18.8 ± 6
Sport volume^a^ [h/week]	7.1 ± 3.8	6.1 ± 3.13	7.7 ± 4.0	7.3 ± 2.9
Borg's scale [1–20]	16.8 ± 1.1	16.4 ± 1.1	17.1 ± 0.9	16.9 ± 0.9
Maximal Heartrate [bpm]	187.1 ± 9.4	191.8 ± 8.6	184.3 ± 8.6	182.9 ± 8.9

DOM, dominant leg; nDOM, non-dominant leg.

^a^ Experience and volume in the main sport.

**Figure 2:**
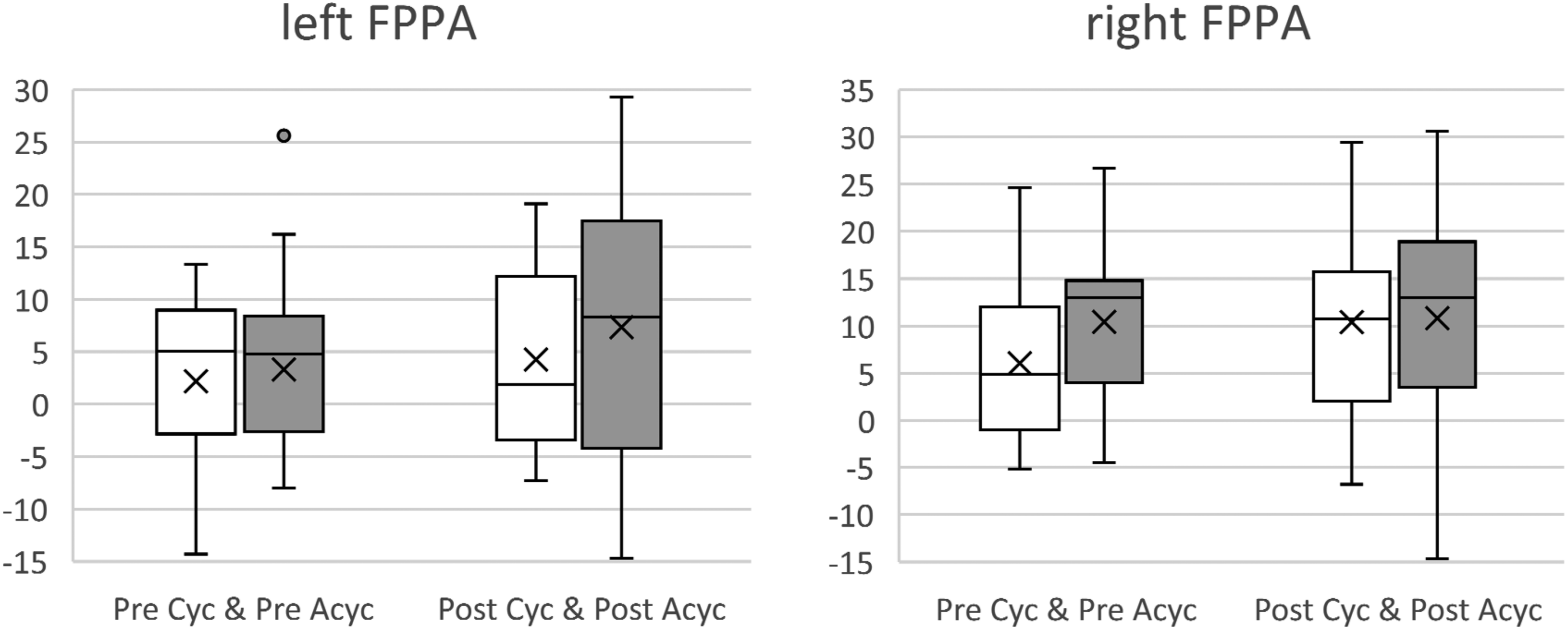
Comparison of cyclic (Cyc) and acyclic (Acyc) sports for the left and right frontal plane projection angle (FPPA) in degree [°] before (pre, white) and after (post, grey) fatigue. The more positive the value, the greater the knee valgus.

Cyclic vs. acyclic sports (see Table 2 and Figure 2): Results show a significant difference between the within-subject factor “time of measurement” (pre-post-fatigue) [*F*(1, 42) = 10.764, *p* = 0.002, np^2 ^= 0.21] and “side” (right vs. left leg) [*F*(1,42) = 12.492, *p* < 0.001, np^2 ^= 0.23]. No significant difference could be found between-subjects (cyclic vs. acyclic) [*F*(1,41) = 0.988, *p* = 0.33].

**Table 2:** Results of the frontal plane projection angle (mean ± standard deviation).

	N	Side	Pre [°]	CI 95% [°]	Post [°]	CI 95% [°]
Overall	43	Left	2.9 ± 9.3	0.0–5.8	6.4 ± 10.5	4.3–10.4
		Right	8.8 ± 8.7	6.1–11.5	10.7 ± 9.9	7.6–13.7
Cyclic	16	Left	2.2 ± 9.7	−3.1–7.5	4.2 ± 9.3	−0.69–9.2
		Right	6.1 ± 8.1	1.7–10.4	10.4 ± 9.7	5.2–15.6
Acyclic	27	Left	3.3 ± 8.9	−0.3–6.9	7.7 ± 11.2	3.3–12.1
		Right	10.4 ± 8.6	7.0–13.9	10.8 ± 9.9	6.8–14.8
Dominant	16	DOM	12.4 ± 8.6	7.9–17.0	13.6 ± 7.8	9.4–17.8
Non-dominant	16	nDOM	4.9 ± 10.3	−0.6–10.42	11.6 ± 10.7	5.7–17.2

**Figure 3:**
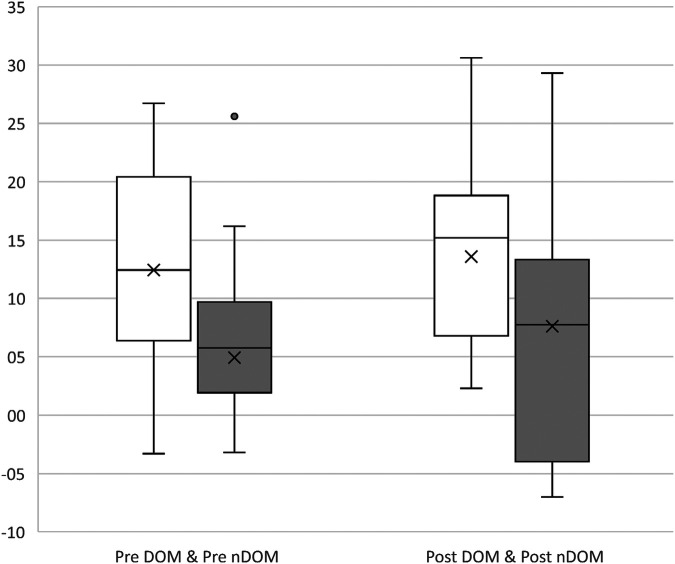
Frontal plane projection angle (FPPA) in degree [°] of the dominant (DOM; kicking leg; white) and non-dominant (nDOM, standing leg; grey) leg before (pre, left) and after (post, right) fatigue. The more positive the value, the greater the knee valgus.

Dominant vs. non-dominant (see Table 2 and Figure 3): Results show a significant difference between the within-subject factor “time of measurement” (pre-post-fatigue) [*F*(1, 15) = 7.06, *p* = 0.02, np^2 ^= 0.32] and a non-significant difference between the “side” (dominant vs. non-dominant) [*F*(1,15) = 4.35, *p* = 0.06, np^2 ^= 0.26].

## 4. Discussion

Depending on the research questions, the results are being discussed in its separate section.

### 4.1. Cyclic vs. acyclic sports

In the present study, there were no kinematic differences between cyclic and acyclic sports in SLDL measured by FPPA. Contrary to the assumption, that acyclic sports show better knee stability, a comparison of the mean values (Figure 2) even shows that the athletes from the acyclic sports in average had a slightly larger knee valgus. The differences in the performance profile of different sports (7, 8) as well as the injury rate (6) actually indicate that athletes from acyclic sports should probably be better trained proprioceptively with regard to stability throughout the SLDL. However, when looking at the movement execution of the SLDL, one quickly realizes that it is not a truly multidimensional movement that challenges the knee joint in all planes. SLDL definitely has a dynamic character due to the drop jump but is relatively linear without the occurrence of greater forces of shear as in cutting movements for example (9, 11). Especially with relatively one-dimensional flexion and extension in the knee joint, which runners in particular experience to a high extent, it is likely that runners have a good knee stabilization ability in SLDL. Thus, a conceivable conclusion could be that the SLDL might not be the appropriate measurement tool to investigate kinematic differences in knee stability between athletes from cyclic and acyclic sports (13, 14). Furthermore, it should be noted that a very wide range of sports can be subsumed under cyclic and acyclic. As far as we know, there is no study that has investigated this research question with regard to the SLDL so far, which is why in the following studies the sample must be forced to be enlarged. Attention must be paid to a more homogeneous division of the two groups (here: acyclic = 27; cyclic = 16) and alternative test methods for the investigation of knee stability differences must be considered (13, 14).

### 4.2. Fatigue and SLDL

The results indicate that fatigue can affect the kinematics of the knee joint. Overall, between pretest and posttest, knee valgus increased significantly with an average of 4.4° for the left leg and 1.9° for the right leg. Furthermore, all subgroup comparisons (cyclic vs. acyclic; dominant vs. non-dominant) also confirmed this result (Figures 2, 3). Many studies confirmed this thesis (27–29), which also seems logical because fatigue is a potential risk factor and the injury rate raises in the second half of matches or in later stages of training (16). However, there several studies that have failed to support this hypothesis (13, 30).

Neuromuscular fatigue is considered to be a state in which the ability to generate and sustain forces is reduced, leading to a decline in muscular performance. Generally, a distinction must be made between central and peripheral fatigue (31). Both are suspected to have an influence on our motor movement pattern and often both manifestations can be found in athletes. The intention of the present study was to investigate peripheral fatigue caused by the shuttle run. While some authors have already investigated the influence of isolated muscular fatigue on knee kinematics (32), we aimed to additionally induce cardiovascular fatigue comparable to that in game sports. It can be assumed that cardiovascular fatigue also leads to a certain amount of neuromuscular fatigue as well, even if in a weaker form compared to the isolated fatigue protocol. Some colleagues (30) point out that especially the fatigue protocol is suspected for studies with missing evidence. In the present study, the shuttle run had to be completed according to the all-out principle. Attention was paid to classic subjective fatigue signs (breathing rate, sweat, skin coloration) to ensure that participants were actually exerting themselves. In addition, pulse monitoring, rate of perceived exertion (RPE) and running distance helped to verify this. Further objective assessments to verify fatigue were not collected, which can be seen as a weakness of the study.

An explanation for the influence of fatigue on kinematics is that fatigue appears to affect both afferent (33) and efferent (34) mechanisms. For example, the firing rate of the motor unit may change and the response of the muscle to stimulation is affected by a decrease in the responsiveness of the motoneuron pool (33). This study shows that the number of norm deviating athletes in terms of comparison with the reference values of Herrington and Munro (23) increased due to the influence of fatigue (28). Based on most studies, the recommendation is to prepare athletes as best as possible for their sportive exposures in order to control fatigue as much as possible, which of course is an important precondition. But fatigue will inevitably occur sooner or later. For this reason, it would also be advisable for fatigue to be more widely used and included in injury prevention through kinematic testing (27, 35). However, even in the case of regular gait analyses, which under certain circumstances do not reveal any norm deviating signs despite clear symptomatic, a follow-up check under the influence of fatigue should be considered.

### 4.3. Dominant vs. non-dominant leg

The replication of the study by Ludwig et al. (20) failed to detect a significant difference between the dominant leg and non-dominant leg. The samples differed with respect to age and size. While Ludwig and colleagues investigated 114 players aged 14.6 ± 1.1 years, the sample here was smaller (*n* = 16) and the age range was larger (25.1 ± 3.4). Notably, the case number severely limits the representativity. Nevertheless, it can be observed (see Figure 3), regardless of the fatigue treatment, in both situations the dominant kicking leg was on average 7.5° (pre fatigue) and 2.0° (post fatigue) more in a valgus axis. The confidence intervals also confirm these mean value trends. An explanation for the non-significant tendency would be the sensorimotor adaptations of the leading stance and kicking leg. According to this, the stance leg is better trained to guarantee stability of the player during passing and kicking in dynamic situations and under the pressure or direct influence of an opponent (36). The profile of the kicking leg, on the other hand, requires a different sensory sensitivity. Here, it is primarily a matter of the correct point of impact of the ball with regard to timing and force. This can lead to sport-specific muscular imbalances, which can possibly promote injuries or stress (37).

On the other hand, the lack of significance might possibly be an indication that a clear separation between a dominant leg and a non-dominant leg is no longer as simple as it was in the past, especially in ambitious amateur and professional sports. Due to the development of the sports game a player is required to be more both-footed than ever before and this is integrated into training of almost all levels (38). Even if the participants of this study did not indicate to be both-footed, the difference might already be less. In addition, even in lower sports levels athletic training has increasingly found its way and is has become part of the weekly training. Furthermore, the higher training age (main sport experience) could possibly resolve in a better muscle status (39) and ability to compensate fatigue. But for a solid basis of these potential interpretations a bigger sample size is necessary.

Kinematic differences between two extremities should be prevented as much as possible (14), both from a sporting point of view (e.g., variability) (38) and from a health point of view (e.g., risk of injury) (36, 37). A follow-up study with players who are actually both-footed could be beneficial to evaluate whether the problem is still relevant or whether training both-footedness is promising in all respects. In addition, a transfer to other sports, such as handball, basketball, would be interesting. The high repetition of jumps in practice and games, which is mostly done with one favored side, possibly leads to adaptations (37) comparable to those in the study of Ludwig et al. (20). Especially, because the single-legged jump and the landing show comparable characteristics, whose main difference can be seen in concentric (jump) and eccentric (landing) muscle work and adaptations.

### 4.4. Limitations

Two-dimensional investigations seem to be outdated in the age of three-dimensional motion analysis via optical or inertial sensory systems. Three-dimensional systems are more accurate, but in practice, for economic reasons (effort-benefit ratio; cost-benefit ratio) and due to the applicability in outdoor settings, two-dimensional analysis is still frequently applied (35) with a well justifiable reliability (40). For this reason, a two-dimensional analysis was deliberately chosen, even though this represents a clear limitation due to the greater measurement inaccuracy.

In general (see Table 2), large confidence intervals can be observed which are due to the high interindividual differences between the athletes. To reduce interindividual differences in the future, it would make sense to perform an intraindividual normalization by calculating the difference between the static FPPA and the dynamic FPPA for every subject.

The SLDL is a convenient test, which can be applied to a wide range of athletes and can also be analyzed with a simple measuring system. It must be remembered that the test does not entirely reflect the loads that occur in sports games during movements in sprinting, with changes of direction and counter-influence at regular intervals. Also it must be mentioned that the sport experience can affect the results of this test. It is and remains a kind of screening test. However, this inversely means that deviations in this type of test might be a clear sign for muscular deficits and of even greater relevance for competition (17).

The motivation to position the athlete's arms on the hips has an intensifying effect on the muscular performance profile, as these have a significant influence on balance (17). The degree of difficulty increases, but the representativity in the sense of external validity decreases since we will not find such a positioning during game sports. Limited to the frontal plane, the SLDL does not provide information about deviating movements, such as rotation of the hip, as a result of muscular fatigue (see Figure 1).

The shuttle run is a test that primarily leads to cardiovascular fatigue. For a better interpretation of the results, an extension with a fatigue protocol that predominantly leads to a muscular fatigue of the knee stabilizing muscles would be beneficial. In addition, objective measures of fatigue would be useful, since for example the Borg scale can be misleading in the evaluation of the state of fatigue.

In conclusion, the calculation of mean values based on several jumps would be better from a statistically point of view, but this was intentionally not done here due to the replication of a study by Ludwig et al. (20). Especially outside the scientific community it is common practice to work with one valid jump due to time limitations.

Finally, the sample size of 43 athletes must be mentioned, which is of course not representative and limits the generalizability of the results.

## 5. Conclusion

Using the SLDL as a measurement tool, no diffserences in knee stability (FPPA) were found between athletes from acyclic (e.g., game sports) and cyclic sports (e.g., athletics). It can be assumed that the SLDL probably is not the adequate method. Differences in knee stability between these groups should be evaluated with other tests (e.g., crossover hop) and fatigue protocols (e.g., repeated jumps) to clarify a potential difference.

Fatigue seems to be able to have a significant influence on knee stability in the frontal axis. Although there does not yet seem to be a clear consensus on the systematic influence of fatigue on knee kinematics (valgization; frontal plane) (14), this point may already be of great importance for prevention, rehabilitation and return to play tests.

The study replication (20) could not confirm its significant difference between the dominant and non-dominant leg for soccer players with regard to the FPPA throughout SLDL. Still, a similar tendency can be seen, which can be explained by the task-specific adaptation of the kicking leg and the stance leg. But overall, the subgroup of soccer players was probably not big enough.

Muscular differences between sports, after fatigue or between the extremities are known and due to neuromuscular adaptations and occur frequently. Consequently, a difference should not necessarily be considered negative, but the prevention of knee stress and knee injuries is complicated and must be analyzed from multiple perspectives. And it must be kept in mind that almost all sports will lead to a certain state of neuro-muscular fatigue, which is why this influencing risk factors should also be increasingly included in movement analyses.

## Data Availability

The raw data supporting the conclusions of this article will be made available by the authors, without undue reservation.
